# The usefulness of the novel 0.018-inch dedicated uneven double-lumen cannula for endoscopic ultrasound-guided hepaticogastrostomy using a 22-gauge needle

**DOI:** 10.1055/a-2802-4849

**Published:** 2026-03-09

**Authors:** Mamoru Takenaka, Hirofumi Kawamoto, Tomohiro Fukunaga, Yuka Sakano, Masayuki Kurimoto, Tae Hoon Lee, Masatoshi Kudo

**Affiliations:** 1 Department of Gastroenterology and Hepatology, Kindai University Faculty of Medicine, Osakasayama, Japan; 2 Department of Internal Medicine, Kawasaki Medical School General Medical Cente, Okayama, Japan; 3 Division of Gastroenterology and Hepatology, Department of Internal Medicine, Soonchunhyang University College of Medicine, Cheonan Hospital, Cheonan-si, Korea; 4 38158 Kindai University Faculty of Medicine Graduate School of Medical Sciences, Osakasayama, Japan

A 78-year-old patient with unresectable hilar cholangiocarcinoma developed malignant hilar biliary obstruction. An inside stent was first placed in the right intrahepatic bile duct, but jaundice persisted. A transpapillary approach to the left duct failed because the guidewire could not traverse the stricture; therefore, endoscopic ultrasound-guided hepaticogastrostomy (EUS-HGS) was performed. To minimize bile leakage, the bile duct was punctured using a 22-gauge EUS-fine needle aspiration needle, and a 0.018-inch guidewire was successfully advanced across the hilar stricture into the common bile duct. However, as the 0.018-inch guidewire alone had limited deliverability, establishing a double-guidewire approach was desirable thereafter.

**Fig. 1:**
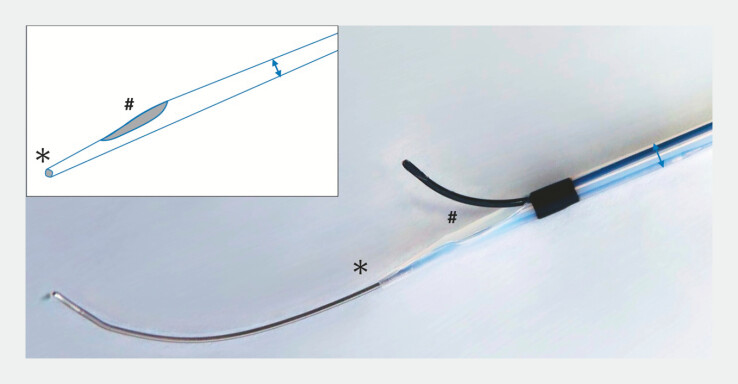
A newly developed 0.018-inch dedicated uneven double-lumen cannula (UDLC) (0.018-UDLC; PIOLAX, Tokyo, Japan) has two lumens (0.018 and 0.035 inch), with the orifice of each lumen being uneven, thereby creating a channel within the tip.

**Fig. 2:**
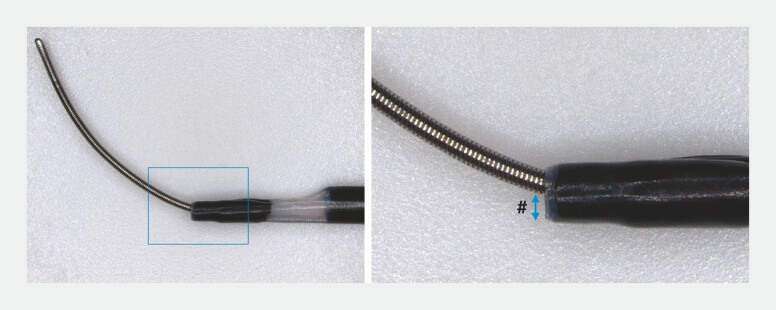
The conventional 0.025-UDLC leaves a gap (#) between the lumen and a 0.018-inch guidewire, reducing pushability and penetration capability. UDLC, uneven double-lumen cannula.

**Fig. 3:**
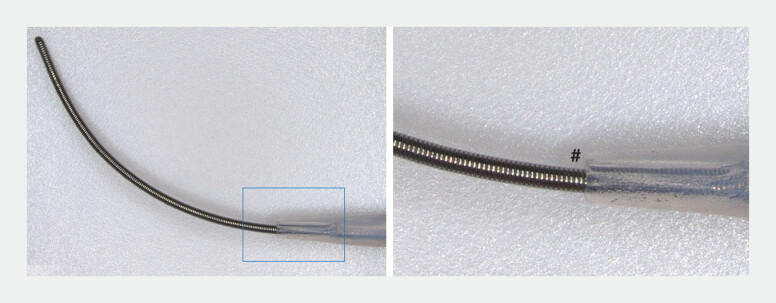
The 0.018-UDLC minimizes the gap (#) between the lumen and a 0.018-inch guidewire, improving tract penetration. UDLC, uneven double-lumen cannula.

The uneven double-lumen cannula (UDLC) has two lumens (0.025 and 0.035 inches), with the orifice of each lumen being uneven, thereby creating a channel within the tip 1 2 3 4 . A newly developed 0.018-inch dedicated UDLC (0.018-UDLC; PIOLAX, Tokyo, Japan) is a modification in which the distal tip lumen has been downsized to 0.018-inch compatibility ( Fig. 1 ). The conventional 0.025-UDLC leaves a gap between the lumen and a 0.018-inch guidewire, reducing pushability and penetration capability ( Fig. 2 ), whereas the 0.018-UDLC minimizes this gap, improving tract penetration ( Fig. 3 ).

**Fig. 4:**
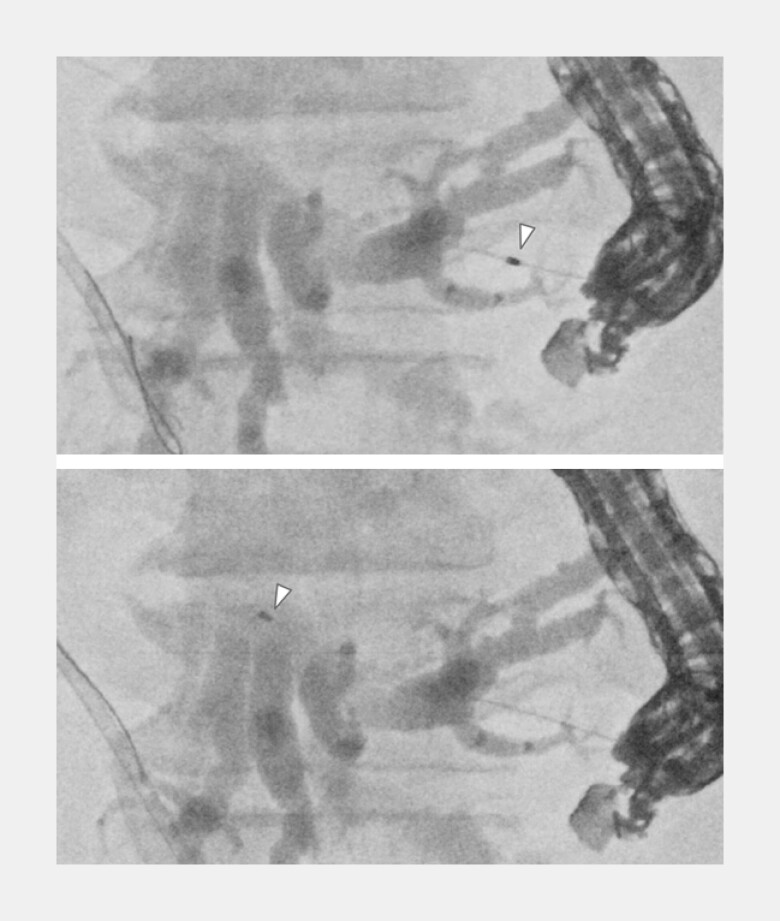
Through the 0.018-inch guidewire, the 0.018-UDLC (arrowhead) successfully penetrated both the gastric and bile duct walls, and the puncture tract was dilated. UDLC, uneven double-lumen cannula.

**Fig. 5:**
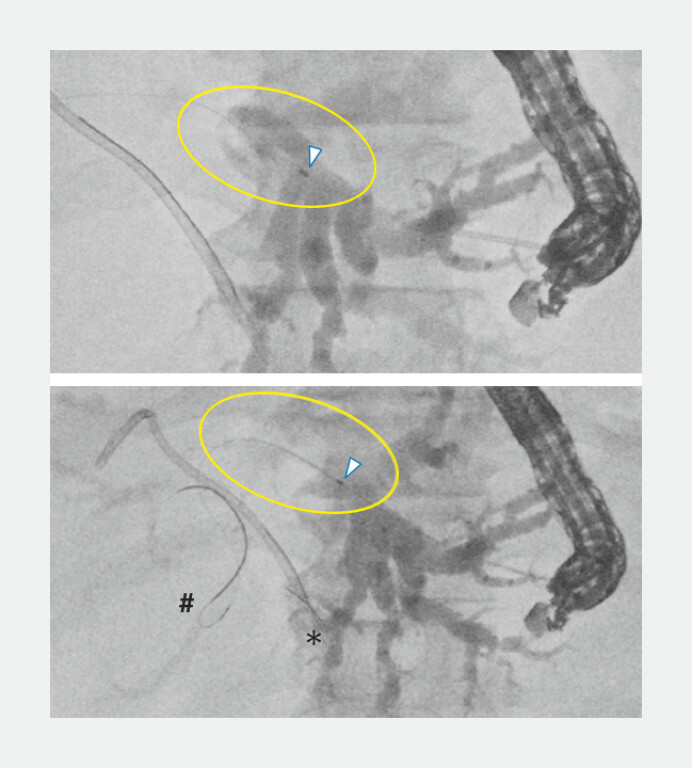
Cholangiography was performed via the proximal lumen (yellow circle area), and a 0.025-inch guidewire from the proximal lumen (#) was advanced beyond the stricture to achieve a double-guidewire situation with a 0.018-inch guidewire from the distal lumen (*).

Through the 0.018-inch guidewire, the 0.018-UDLC successfully penetrated both the gastric and bile duct walls, and the puncture tract was dilated ( Fig. 4 ). Cholangiography was performed via the proximal lumen, and a 0.025-inch guidewire from the proximal lumen was advanced beyond the stricture to achieve a double-guidewire situation with a 0.018-inch guidewire from the distal lumen 5 ( Fig. 5 ).

Subsequently, a 0.025-inch dedicated laser-cut self-expandable metal stent designed for EUS-HGS (Covered Bile Rush Advance; PIOLAX, Tokyo, Japan) was successfully deployed over the 0.025-inch guidewire ( Video 1 ).

The utility of a novel 0.018-inch dedicated, uneven double-lumen cannula for EUS-HGS in malignant hilar biliary obstruction. This newly developed 0.018-UDLC enables tract dilation, cholangiography, and double-guidewire creation using a single device, representing a practical innovation that may enhance future EUS-HGS procedures. EUS-HGS, endoscopic ultrasound-guided hepaticogastrostomy.Video 1

This newly developed 0.018-UDLC enables tract dilation, cholangiography, and double-guidewire creation using a single device, thereby enhancing the potential for interventional EUS procedures using a 22-gauge needle.

Endoscopy_UCTN_Code_TTT_1AS_2AH
